# Rhodopsin Expression Level Affects Rod Outer Segment Morphology and Photoresponse Kinetics

**DOI:** 10.1371/journal.pone.0037832

**Published:** 2012-05-25

**Authors:** Clint L. Makino, Xiao-Hong Wen, Norman A. Michaud, Henry I. Covington, Emmanuele DiBenedetto, Heidi E. Hamm, Janis Lem, Giovanni Caruso

**Affiliations:** 1 Department of Ophthalmology, Massachusetts Eye and Ear Infirmary, Harvard Medical School, Boston, Massachusetts, United States of America; 2 Department of Mathematics, Vanderbilt University, Nashville, Tennessee, United States of America; 3 Department of Pharmacology, Vanderbilt University, Nashville, Tennessee, United States of America; 4 Department of Ophthalmology, Program in Genetics, Program in Neuroscience, Program in Cell, Molecular and Developmental Biology, Tufts University School of Medicine, Boston, Massachusetts, United States of America; 5 Construction Technologies Institute, National Research Council, Rome, Italy; University of Oldenburg, Germany

## Abstract

**Background:**

The retinal rod outer segment is a sensory cilium that is specialized for the conversion of light into an electrical signal. Within the cilium, up to several thousand membranous disks contain as many as a billion copies of rhodopsin for efficient photon capture. Disks are continually turned over, requiring the daily synthesis of a prodigious amount of rhodopsin. To promote axial diffusion in the aqueous cytoplasm, the disks have one or more incisures. Across vertebrates, the range of disk diameters spans an order of magnitude, and the number and length of the incisures vary considerably, but the mechanisms controlling disk architecture are not well understood. The finding that transgenic mice overexpressing rhodopsin have enlarged disks lacking an incisure prompted us to test whether lowered rhodopsin levels constrain disk assembly.

**Methodology/Principal Findings:**

The structure and function of rods from hemizygous rhodopsin knockout (R+/−) mice with decreased rhodopsin expression were analyzed by transmission electron microscopy and single cell recording. R+/− rods were structurally altered in three ways: disk shape changed from circular to elliptical, disk surface area decreased, and the single incisure lengthened to divide the disk into two sections. Photocurrent responses to flashes recovered more rapidly than normal. A spatially resolved model of phototransduction indicated that changes in the packing densities of rhodopsin and other transduction proteins were responsible. The decrease in aqueous outer segment volume and the lengthened incisure had only minor effects on photon response amplitude and kinetics.

**Conclusions/Significance:**

Rhodopsin availability limits disk assembly and outer segment girth in normal rods. The incisure may buffer the supply of structural proteins needed to form larger disks. Decreased rhodopsin level accelerated photoresponse kinetics by increasing the rates of molecular collisions on the membrane. Faster responses, together with fewer rhodopsins, combine to lower overall sensitivity of R+/− rods to light.

## Introduction

The outer segment of a retinal rod is an elaborate sensory cilium that is highly specialized for transducing light into an electrical signal, reviewed in [1]. Within the outer segment, photoexcited rhodopsin promotes nucleotide exchange on the G protein transducin, whose alpha-subunit then stimulates the hydrolysis of cGMP by a phosphodiesterease, PDE. Cyclic nucleotide gated ion channels close and the ensuing hyperpolarization spreads passively to the opposite end of the rod, where it alters synaptic transmission to second order neurons, reviewed in [2], [3].

To capture photons efficiently, the outer segment interposes into the optical path up to several thousand disks, whose membranes are densely packed with rhodopsin. Depending upon the number of disks and their diameter, an outer segment contains ten million to a billion rhodopsin molecules. Outer segment girth varies greatly across species, particularly in fish where they may range from less than 1 µm to nearly 20 µm in diameter [4], [5]. The edges of disks in some species are scalloped, while those in other species are deeply cleft by one or more incisures. Incisures are typically aligned in consecutive disks, creating axial passageways that enhance the longitudinal diffusion of soluble substances in phototransduction. Furthermore, the outer segments in some species extend 200 µm away from the mitochondria in the inner segment [6], so incisures are likely to play an important role in maintaining metabolic homeostasis.

The mechanisms that determine disk morphology are not known. Rhodopsin is essential for disk formation because in homozygous rhodopsin knockout rods, rod outer segments (ROSs) are not elaborated [7], [8]. Overexpression of rhodopsin in transgenic mouse rods [9] causes disk enlargement [10] suggesting that disk size depends upon the amount of rhodopsin transported from the inner segment where it is synthesized to the base of the outer segment, the site of nascent disk formation, reviewed in [11]. The single incisure found in normal disks disappears in the oversized disks of rods overexpressing rhodopsin, perhaps because the levels of structural proteins used to stabilize the hairpin turn at the disk rim and the incisure have remained constant and are no longer adequate to meet the structural demand. If these hypotheses were true, then a reduction in rhodopsin production would result in diminutive disks with a surplus of structural protein and a more extensive incisure. As a test, we studied the rods of hemizygous rhodopsin knockout (R+/−) mice, which express half the normal amount of rhodopsin [8], [12]. The predictions were borne out; R+/− rods did form smaller disks with a longer incisure.

## Results and Discussion

**Figure 1:**
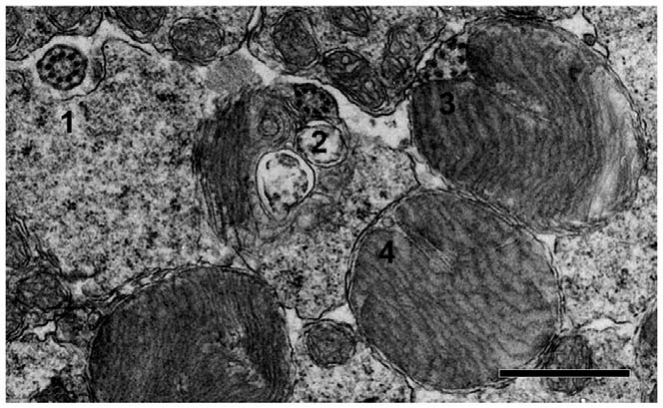
Cross sections of WT rods. 1, transition zone where the axoneme appeared to be separated from the inner segment; 2, outer segment with nascent disks; 3, at a level more distal to the inner segment, where disks were full sized; 4, further distal where a tubular structure or a vesicle was the last remnant of the axoneme. Scale bar 1 µm.

R+/− mouse rods, expressing half the normal amount of rhodopsin, had outer segments that differed from those of WT in three ways. First, R+/− ROS were elliptical rather than circular in cross section. Second, the surface area of the R+/− disk was smaller. Third, the R+/− incisure was more extensive and bisected the disk. An elliptical shape could result from failure to section a right circular cylinder perpendicular to its long axis or from proper sectioning of an elliptical cylinder. To distinguish between the two possibilities, we sectioned the globe tangentially at a level where inner segments were prevalent, because at that level adjacent cells tended to retain a more orderly alignment. In the murine retina, the distance of the ROS base from the outer nuclear layer is not uniform. Thus in the same field, some rods were sectioned at the inner segment while others were sectioned at the outer segment. Between the inner and outer segment, the axoneme or connecting cilium is round in cross section (cell#1 in **Fig. 1**), so it served as a convenient reference. Basal disks evaginating from the axoneme (cell#2 in **Fig. 1**) did not reach the full diameter and were excluded from consideration. Distally, as disks became full sized, the axoneme transitioned to a more triangular structure with microtubules splayed out along its sides (cell#3 in **Fig. 1**, see also [13], [14], [15]). A slit-like incisure penetrated deeper into the disk from the apex of the triangle, opposite the base of the triangle that was continuous with the disk's outer rim. Complex fimbriae that appear at the apex of the infolding in osmium fixed disks [16], [17] were not observed. The triangular wedge flattened in disks more distal to the inner segment. The doublets of microtubules reduced to singlets [14], [18] and then dropped out at variable distances from the inner segment. As the wedge became minimal (cell#4 in **Fig. 1**), one or more tubular structures sometimes occupied that space [13], [15]. In other rods, such structures were often missing, perhaps because microtubules succumbed to disruption during tissue preparation [19].

Rod disks in mouse are typically punctuated by a single incisure [16], [20]. Cone outer segments are smaller in diameter, taper and their disks are often split by multiple incisures [21]. By selecting profiles with a single incisure, we minimized cone inclusion in estimating the mean dimensions of rods. The low, ∼3% frequency of cones also favored cone exclusion [21]. Profiles lacking an incisure could not be identified unambiguously as rods and were not pursued further.

**Figure 2:**
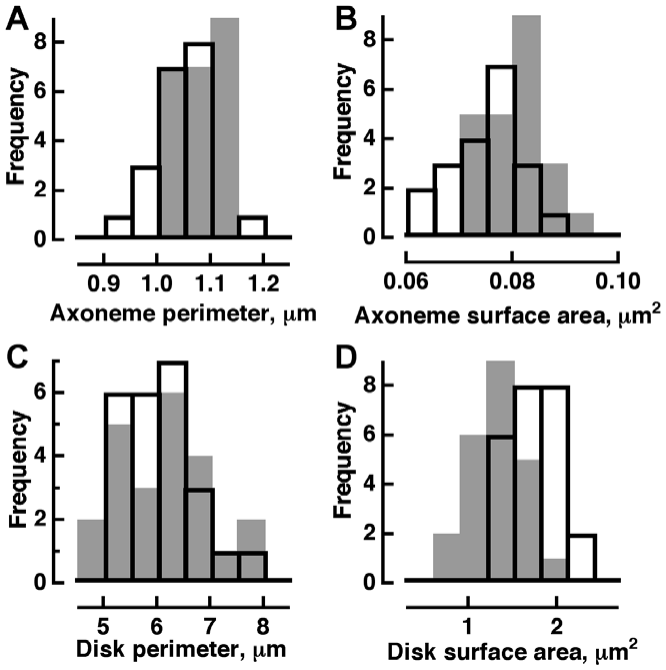
Sizes and shapes of axonemes and disks in R+/− and WT rods from central retina. WT, open bars; R+/−, gray bars. **A, B,** Very minor increase in apparent R+/− axoneme size. Circularity, defined as 4π (area)/circumference^2^, was 0.876±0.005 for WT (mean ± SEM, n = 20) and 0.883±0.006 for R+/− (n = 15). Roundness = minor axis/major axis was 0.88±0.02 for WT and 0.88±0.01 for R+/−. For circular profiles, both parameters take values of 1.0. Mean perimeter for WT axoneme was 1.04±0.01 µm, while for R+/− it was 1.08±0.01 µm (p<5e-3). Mean cross sectional surface area for WT axoneme was 0.075±0.001 µm^2^, while for R+/−, it was 0.082±0.002 µm^2^ (p<5e-3). **C,D,** Reduced surface area of R+/− disks without a change in perimeter. Mean disk perimeters for WT and R+/− were 6.0±0.1 µm and 6.2±0.3 µm, respectively (n.s.). Values for surface area refer to one of the two disk faces. Mean surface area for WT disks was 1.69±0.06 µm^2^ (n = 21), while for R+/− it was 1.33±0.09 µm^2^ (n = 14, p<3e-5).

In our samples of WT and R+/− axonemes matched for circularity, the latter were slightly enlarged in perimeter and cross sectional area by 4% and 9%, respectively (**Fig. 2A, B**). The differences were attributed to experimental error in measurement because contours were less clearly defined for many mutant axonemes in our micrographs. The same measurement error became insignificant for the considerably larger disks, below. Axoneme diameters, calculated from the measurements of area were 0.31±0.02 µm for WT and 0.32±0.02 µm for R+/−. According to the literature, rod axonemes are ∼0.25 µm in diameter [22]; tapering slightly from 0.23 µm near the inner segment to 0.28 µm at the base of the outer segment [23]. Our values were overestimates because some slightly oblique sections were included. To improve accuracy, we found the diameter of the largest circle that would fit within each axoneme profile: 0.282±0.003 µm for WT and 0.299±0.003 µm for R+/−.

**Figure 3:**
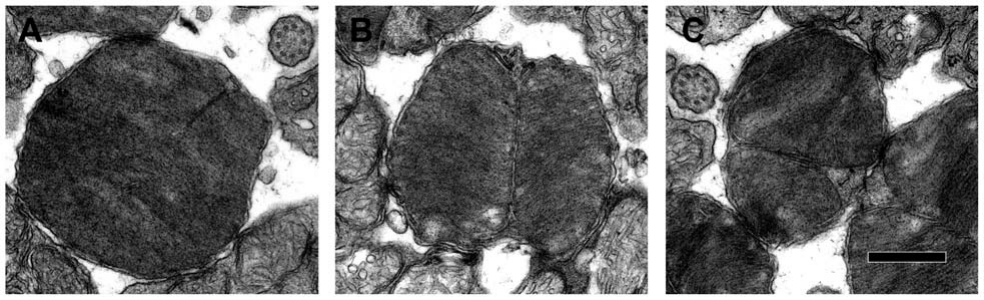
Disk surfaces of WT (A) and R+/− (B, C). The R+/− disk may be divided symmetrically or asymmetrically by the incisure. The axonemes in **A** and **C** had roundness values of 0.89 and 0.94, respectively, while the outer segments had roundness values of 0.95 and 0.71, respectively. The outer segment in **B**, which lacked a reference axoneme, had a roundness value of 0.92. Scale bar 0.5 µm.

After using axonemes to ensure equality in the angle of tissue sectioning, R+/− rod outer segments were indeed less round than those of WT (e.g., **Fig. 3C**) as judged by circularity: WT 0.874±0.005 versus R+/− 0.823±0.009 (p<2e-3) and roundness: WT 0.83±0.02 versus R+/− 0.75±0.02 (p<8e-3). Thus R+/− ROSs were elliptical cylinders, in contrast to WT ROSs which were right circular cylinders.

The average disk diameter, calculated from the cross sectional area was 1.47 µm for WT. Here again, imprecision in the angle of sectioning caused this value to be slightly high. Refining the estimate, as described above for axonemes, reduced the diameter to 1.37±0.03 µm (n = 21), which matched the mean value drawn from a larger sample over a greater range of regions across the retina and viewed at different distances from the outer limiting membrane, 1.36±0.01 µm (n = 142 rods) and fell within the range of 1.35 to 1.44 µm reported previously for mouse [10], [21], [24], [25], [26]. The “equivalent diameter” for R+/− ROSs, computed from their mean cross sectional area (**Fig. 2D**), was 1.30 µm. After multiplying the R+/− equivalent diameter by the ratio of the two different estimates for WT outer segment diameter, (1.30 µm)(1.37/1.47) = 1.21 µm, the true R+/− ROS area was calculated to be 1.15 µm^2^, about ∼20% lower than that for WT, 1.47 µm^2^. The smaller size of R+/− disks suggests that disks form at fixed intervals and that the size of a nascent disk was determined by the amount of rhodopsin delivered to the outer segment within that interval.

The change in disk shape was characterized by normalizing the roundness value for R+/− ROSs by that for WT, 0.75/0.83 = 0.90, and then solving for the area of an ellipse = π(major radius)(minor radius). The major radius was 0.64 µm and the minor radius was 0.57 µm. Evidence will be presented below that the distortion in disk shape arose from a surplus of structural proteins that stabilizes the disk rim.

A striking feature of the R+/− rod was the prominence of its incisure. In 574 out of 775 rods in which an incisure was resolved, the incisure completely transected the disk, almost always spanning its minor axis (**Fig. 3B,C**). In the remainder of the rods, the incisure was elongated but did not quite make it all the way across the disk. The incisure in WT rods rarely divided the disk (19 out of 835 rods). In the few cases where it did, the division was asymmetric, i.e., chord length was less than ROS diameter. In WT rods, the incisure penetrated 0.44±0.02 (n = 21) of the distance across the disk.

Interestingly, the perimeter (distance around the outer disk edge plus twice the incisure length if the incisure does not split the disk in two) of the R+/− disks was normal (**Fig. 2C**) suggesting that in mouse, shrinkage of disk circumference permitted expansion of the incisure. The disk margin and its perimeter are lined with filaments [27], [28], [29], [30] that are thought to organize the membrane at the disk edge and along the incisure into hairpin turns and to stabilize the separation of consecutive disks. Fewer structural proteins were needed to line the outer edge of a small R+/− disk, so the “excess” was incorporated into the incisure. Conversely, the incisure seems to shorten in enlarged disks of mice overexpressing rhodopsin [10]. In some sense, the incisure may buffer structural proteins, affording a safety margin for individual rods facing daily variations in rhodopsin expression and nascent disk size during outer segment renewal. A naturally occurring parallel appears in cat cone, where a single incisure in basal disks lengthens in more distally located disks as the outer segment tapers. At the outer segment tip, the incisure extends completely across the disk and the cross sectional profile becomes more elliptical [13]. Besides distal cat cone disks, the photoreceptor disks of pigeon [31] and Tokay gecko [32] are transected by one or more incisures.

**Figure 4:**
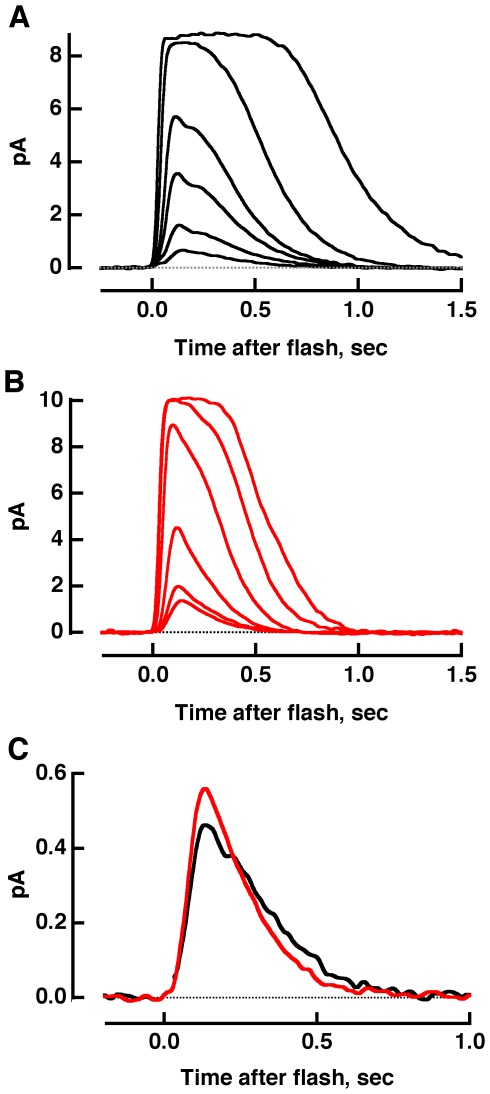
Flash responses from WT and R+/− rods. Each trace was an average obtained from 17 to 45 rods, where the contribution from each rod was itself an average of at least three trials for bright flashes and as many as 180 trials for dim flashes. WT includes some results from [50]. Mean flash strengths for WT (**A**) were: 9, 18, 40, 70, 256 and 1111 photons µm^−2^, while for R+/− (**B**), they were: 18, 37, 94, 337, 718 and 1529 photons µm^−2^ at 500 nm. **C.** Faster single photon response recovery in R+/− rods. The dim flash response, with an amplitude less than a fifth of the maximum, has the same kinetics as the single photon response. So dim flash responses were scaled to the amplitude of the single photon response for each rod, found from the ratio of the ensemble variance to the mean, and averaged for 10 WT (black) and 17 R+/− (red) rods. A flash artifact was removed from the WT response.

The impact of reduced rhodopsin expression and subsequent changes in disk structure on phototransduction was explored in single cell recordings. Flash responses from R+/− rods had faster recovery kinetics than those of WT rods as reported previously [8], [12], however, for the rods in the present study, a faster rising phase was not observed. The basis for the phenotypic variation from the prior studies is not known but may have been caused by genetic drift. Single photon response amplitude was normal, yet R+/− rods were approximately half as sensitive as WT rods. With half as many rhodopsins, R+/− rods suffered from a lowered capacity to capture photons. Single photon responses in R+/− also had a smaller integration time with a faster time constant for response recovery (**Fig. 4** **, Table S1**).

The “missing” rhodopsin in R+/− disks is partially replaced with phospholipid [12] probably because when rhodopsin synthesis declines, shipments of rhodopsin to the ROS include greater ratios of lipid and/or more lipid accompanies transport of non-rhodopsin containing shipments cf. [33], [34], [35], [36]. With a higher phospholipid to rhodopsin ratio in R+/− ROSs, the accelerated flash response kinetics [8], [12], [37] were initially attributed to quicker collision rates between membrane proteins [12]. Lower rhodopsin expression relieved membrane crowding and enhanced the lateral diffusion of key phototransduction proteins on the disk membrane [38]. That interpretation was questioned when it was later discovered that R+/− ROS diameter was smaller [37]. Lower aqueous volume between the disks would accelerate any changes in cGMP concentration during phototransduction [39]. Moreover, neither study considered the expanded incisure. Yet heat flow modeling indicates that incisures can slow the apparent lateral diffusion of membrane proteins [40]. Incisures also promote the longitudinal diffusion of soluble substances such as cGMP and Ca^2+^ and thereby affect the gain and reproducibility of the single photon response [41]. In the present study, R+/− ROS girth and hence, cytoplasmic volume were not as small as reported in [37]. The discrepancy may have been related to our finding that ROS shape changed from a right circular cylinder to an elliptical cylinder. For all these reasons, it was important to revisit the basis for accelerated R+/− photon response kinetics.

A spatially resolved model for phototransduction [41], [42] was used to evaluate the effects of the changes in rhodopsin expression and outer segment morphology on the photon response. For the simulations, the R+/− outer segment was taken to be a circular cylinder of reduced diameter (see above). Additional measurements to determine the dimensions of the aqueous spaces were generally in agreement with those from vitrified samples subjected to cryoelectron tomography [43]. The distance from disk rim to plasma membrane was measured for 14 to 99 disks in each of 19 WT rods and the ensemble average found to be 14.8±0.5 nm, similar to the value of 17 nm reported by [43]. The separation between adjacent disks, measured interior to the hairpin turns in 10 WT ROSs was 8.8±0.3 nm, somewhat less than that reported by [43], possibly because of our selective sampling (see Methods). For a disk to disk repeat distance of 32.3±0.3 nm (n = 127 WT rods, includes results from [10]), the thickness of the disk was 32.3−8.8 = 23.5 nm. Measurements of the corresponding parameters in R+/− rods yielded values that were not significantly different from those of WT rods. The incisure, with a width of 10.9±0.4 nm (n = 43 WT rods), penetrated (0.44)(1.37 µm) = 0.60 µm across the WT disk and 1.14 µm across the R+/− disk.

**Figure 5:**
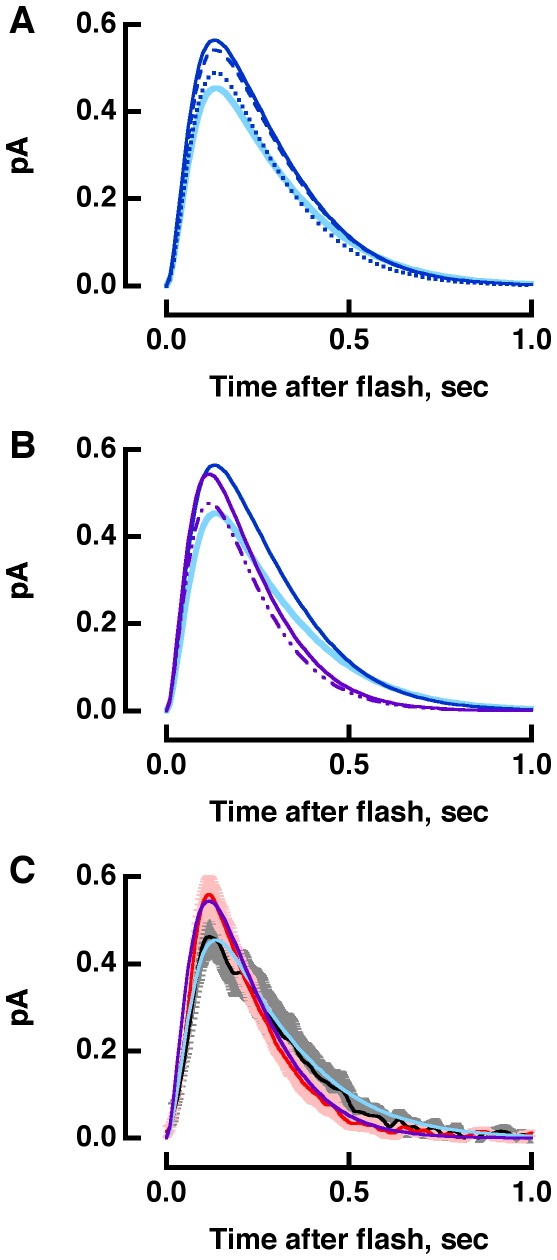
Modeling the accelerated single photon response in R+/− rods. **A.** Simulations of the WT single photon response (continuous, light blue) with a 20% lower ROS volume (dashed, royal blue), a 90% longer incisure (dotted, royal blue) or both (continuous, royal blue). **B**. Accelerated response recovery and reduction in amplitude upon decreasing ROS volume, lengthening the incisure and increasing rhodopsin shutoff and transducin/PDE shutoff by 1.3-fold (dash-dot-dot, violet). Inclusion of a 1.3-fold faster transducin activation along with all other factors enlarged the response (continuous, violet). Royal blue and light blue traces are reproduced from **A**.**C**. Comparison of modeled responses for WT (light blue) and R+/− (violet) to experimentally observed single photon responses (WT in black, R+/− in red from **Fig. 4C**, with error bars showing SEM in gray and pink, respectively).

The model enabled us to analyze piecemeal the effects of each perturbation on the flash response. Lengthening the incisure improved longitudinal diffusion of aqueous solutes but the effect was modest because there was only one incisure and its width was so thin. Decreased ROS volume produced a larger increase in the amplitude of the single photon response due to the greater change in concentration of cGMP for a given number of active PDEs (**Fig. 5A**). Normal expression levels of transducin, rhodopsin kinase and arrestin in R+/− rods [8], [12] meant that their respective concentrations actually increased by the ratio of the WT disk surface area to that of R+/−, (1.47 µm^2^)/(1.15 µm^2^) = 1.28-fold. Consequently membrane proteins collided with one another more frequently: photoexcited rhodopsin with rhodopsin kinase and transducin, and transducin with PDE and RGS9 complex. After incorporating the effects of decreased volume, longer incisure and faster cascade shutoff, the single photon response amplitude was reduced to slightly less than normal size and the recovery quickened (**Fig. 5B**). Increasing the collision rates between photoexcited rhodopsin and transducin, as well as between transducin and PDE enlarged the response (**Fig. 5B**). Although the observed R+/− response was slightly larger than normal, the difference was close to the resolution of the experimental measurement (**Fig. 5C**, see also **Table S1**). The reduction in rhodopsin expression also relieved membrane crowding, as indicated by the decreased specific absorbance and the increased phospholipid to rhodopsin ratio of R+/− ROSs [8], [12], however, additional adjustments to the reaction rates were not required in these simulations. Thus the spatially resolved model of phototransduction confirmed that faster rates of molecular collisions on the disk membrane were most important in accelerating the R+/− photoresponse. In the future, it would be interesting to explore how incisure length and ROS diameter affect the translocation rates of certain phototransduction proteins between outer and inner segments after exposure to very bright light, reviewed in [2] and the overall effect of reduced rhodopsin expression on visual behavior.

Rats subjected to dietary restriction of vitamin A (but provided a source of retinoic acid) experience a decline in opsin levels. Disk size diminishes in the rods, consistent with our results on R+/− mouse rods, yet the rat disks remain circular and the incisure does not split the disk surface [44]. Another important difference is that the packing density of rhodopsin in the disk membrane remains constant in vitamin A deprived rats, whereas it is reduced in R+/− mouse rods [8], [12]. Therefore, it is likely that the effects of vitamin A deficiency are not specific to opsin expression, rather the condition impairs the syntheses of membrane as well as other proteins necessary for disk morphogenesis.

Raising the expression level of opsin drives disk expansion but can lead to disruption in the ROS and rod degeneration [9], [45]. Thus any substantial changes must be accompanied by increased expression of other proteins in order to build a sound structure with reasonable response amplification and kinetics. An extensive endoplasmic reticulum is required for the daily synthesis of tens of millions of rhodopsin copies needed for ROS turnover in the largest photoreceptors. An expanded ROS volume would support greater ion fluxes, that along with the production of cGMP, would place tremendous metabolic demands on the rod calling for a proliferation of mitochondria, reviewed in [46]. This model explains why only rods with large inner segments are capable of constructing and maintaining large outer segments. Specification of inner and outer segment size may involve regulation of Crumbs protein expression and activity [47], [48]. With the advent of genetic approaches towards correcting degenerative, disease-causing mutations in rhodopsin, reviewed in [49], it becomes increasingly important to consider the effect of opsin expression level on rod function and viability.

## Materials and Methods

### Animal model

This study adhered to the recommendations in the Guide for the Care and Use of Laboratory Animals of the National Institute of Health. Protocols 95-06-006 and B2009-22 were approved by the Institutional Animal Care and Use Committees of the Massachusetts Eye and Ear Infirmary and Tufts University School of Medicine, respectively. Two R+/− mice [8], aged seven weeks and two WT mice, aged nine weeks were dark adapted overnight. Their eyes were removed under infrared illumination and immersed in modified Karnovsky's fixative: 2.5% glutaraldehyde, 2% formaldehyde, 0.08 M CaCl_2_ in 0.1 M cacodylate buffer, at 4°C for 15 min. Then under normal room lighting, the anterior segments were removed and fixation of the eyecups continued for approximately 24 hours. After washing with 0.1 M cacodylate, eyes were post fixed in 2% aqueous OsO_4_, dehydrated with a graded series of ethanol and then propylene oxide, embedded in Epon (Tepon resin, Tousimis, USA) and cured for 48 hrs at 60°C. Tangential sections of retina, 70 to 90 nm thick, were stained with uranyl acetate and Sato's lead stain and mounted on a Philips CM-10 electron microscope. Micrographs at magnifications ranging from 15000× to 34000× were digitally captured as 3056 by 3186 pixel images using an SIA camera (Duluth, GA) with Maxim DL5 software (Diffraction Limited, Ottawa, Canada). Some micrographs were captured on film and digitally scanned. The plasma membranes surrounding a ROS and that of a neighboring axoneme were traced and assessed for circularity and roundness with ImageJ 1.42q (NIH). Micrographs in which circularity of the axoneme was <0.85 were rejected. Disk perimeter and area were determined from samples obtained from central retina, after tracing the outline of the disk and its incisure. The distances separating the membranes between consecutive disks and between disk and plasma membrane were determined from longitudinal sections of retinas from additional WT mice that were processed separately. Measurements were restricted to areas where the disks were regularly spaced and were not swollen. Comparisons were made with a two-tailed t-test.

### Physiology

Flash responses were recorded from single rods of 12 WT and 7 R+/− mice, 5–8 weeks old. Retinas were dissected under infrared light and stored on ice in Leibovitz's L-15 medium (Invitrogen, Grand Island, NY) containing 0.1 mg ml^−1^ bovine serum albumin (Fraction V, Sigma, St. Louis, MO) and 10 mM glucose. A piece of retina was chopped finely in an enriched, bicarbonate buffered Locke's solution containing (mM): 139 Na^+^, 3.6 K^+^, 2.4 Mg^2+^, 1.2 Ca^2+^, 123.3 Cl^−^, 20 HCO_3_^−^, 10 HEPES, 3 succinate, 0.5 L-glutamate, 0.02 EDTA and 10 glucose, 1% (v/v) minimal essential medium amino acids (Invitrogen), 1% (v/v) basal medium Eagle vitamins (Sigma), and DNase I (Type IV-S, Sigma). The tissue was then transferred into a recording chamber and perfused constantly with the enriched Locke's solution equilibrated with 95% O_2_/5% CO_2_. A rod outer segment was sucked into a silanized glass electrode that was filled with (mM): 140 Na^+^, 3.6 K^+^, 2.4 Mg^2+^, 1.2 Ca^2+^, 145.8 Cl^−^, 10 HEPES, 0.02 EDTA and 10 glucose (pH 7.4). Chamber temperature was controlled to be 37±0.5°C. Light stimuli from a xenon arc light source passing through a six cavity interference filter (500 nm, Omega Optical, Brattleboro, VT) and neutral density filters were presented as a 23 msec flash. Photocurrent was measured with an Axopatch 200A amplifier (Axon Instruments, Union City, CA), filtered at 30 Hz (−3 dB, 8-pole Bessel, Frequency Devices, Haverhill, MA) and digitized at 400 Hz by Pulse/PulseFit (version 8.07, HEKA Elektronik, Germany). Data were analyzed off-line using Igor Pro (version 5.03, WaveMetrics, Inc., Lake Oswego, OR) with 12 Hz digital filtering. Records were not corrected for the delay introduced by low pass filtering.

## Supporting Information

Table S1 **Flash response parameters of WT and R+/− rods from single cell recordings.** Mean ± SEM, n. The i_0.5_, which is the flash strength at 500 nm that produced a half maximal response, varies as the multiplicative inverse of sensitivity. The single photon response parameters were determined from dim flash responses, whose amplitudes were less than a fifth of the maximum. Amplitude was determined as the ratio of the ensemble variance to the mean of the responses. Time to peak was measured from midflash to the peak of the response. Integration time was taken as the time integral under the response divided by the amplitude. Recovery time constant describes the fit of the final falling phase of the response to an exponential function. The maximal response amplitude provided a crude measure of the amplitude of the circulating current in darkness. The saturation time constant estimates the dominant time constant for photoresponse recovery. It was determined as the slope of the relation between saturation time and natural logarithm of the flash strength, for bright flashes. Saturation time was measured from midflash to 20% recovery of the response. In general, these values corresponded well to those of the average responses in **Fig. 4C**, except for time to peak, for which the latter showed similar values for WT and R+/−. WT parameters include results from [50].
(DOCX)
