# Supplementary material for: Rhodopsin Expression Level Affects Rod Outer Segment Morphology and Photoresponse Kinetics
Source: PLoS One. 2012 May 25;7(5):e37832. doi: 10.1371/journal.pone.0037832 (PMC3360601; doi:10.1371/journal.pone.0037832)
Supplement: Table S1 — Flash response parameters of WT and R+/− rods from single cell recordings. Mean ± SEM, n. The i0.5, which is the flash strength at 500 nm that produced a half maximal response, varies as the multiplicative inverse of sensitivity. The single photon response parameters were determined from dim flash responses, whose amplitudes were less than a fifth of the maximum. Amplitude was determined as the ratio of the ensemble variance to the mean of the responses. Time to peak was measured from midflash to the peak of the response. Integration time was taken as the time integral under the response divided by the amplitude. Recovery time constant describes the fit of the final falling phase of the response to an exponential function. The maximal response amplitude provided a crude measure of the amplitude of the circulating current in darkness. The saturation time constant estimates the dominant time constant for photoresponse recovery. It was determined as the slope of the relation between saturation time and natural logarithm of the flash strength, for bright flashes. Saturation time was measured from midflash to 20% recovery of the response. In general, these values corresponded well to those of the average responses in Fig. 4C , except for time to peak, for which the latter showed similar values for WT and R+/−. WT parameters include results from [50]. (DOCX) [file pone.0037832.s001.docx]

**Table S1**

|  | WT | Rho+/- |
| --- | --- | --- |
| i _0.5_, photons μm^-2^ | 46 ± 2, 72 | 85 ± 5, 44 |
| Circulating current, pA | 9.1 ± 0.3, 74 | 9.4 ± 0.3, 49 |
| Single photon response  Amplitude, pA | 0.51 ± 0.05, 10 | 0.58 ± 0.04, 17 |
| Time to peak, ms | 145 ± 5, 46 | 131 ± 3, 28 |
| Integration time, ms | 328 ± 18, 46 | 231 ± 9, 28 |
| Recovery time constant, ms | 234 ± 16, 46 | 151 ± 12, 28 |
| Saturation time constant, ms | 274 ± 11, 60 | 148 ± 9, 43 |
